# Assessment of 3D Visual Discomfort Based on Dynamic Functional Connectivity Analysis with HMM in EEG

**DOI:** 10.3390/brainsci12070937

**Published:** 2022-07-18

**Authors:** Zhiying Long, Lu Liu, Xuefeng Yuan, Yawen Zheng, Yantong Niu, Li Yao

**Affiliations:** 1 State Key Laboratory of Cognitive Neuroscience and Learning & IDG/McGovern Institute for Brain Research, Beijing Normal University, Beijing 100875, China; liulu94@meituan.com (L.L.); 202121061103@mail.bnu.edu.cn (X.Y.); 202021061111@mail.bnu.edu.cn (Y.N.); yaoli@bnu.edu.cn (L.Y.); 2 School of Artificial Intelligence, Beijing Normal University, Beijing 100875, China; 201831210013@mail.bnu.edu.cn

**Keywords:** EEG, HMM, dynamic functional connectivity, 3D visual discomfort

## Abstract

Stereoscopic displays can induce visual discomfort despite their wide application. Electroencephalography (EEG) technology has been applied to assess 3D visual discomfort, because it can capture brain activities with high temporal resolution. Previous studies explored the frequency and temporal features relevant to visual discomfort in EEG data. Recently, it was demonstrated that functional connectivity between brain regions fluctuates with time. However, the relationship between 3D visual discomfort and dynamic functional connectivity (DFC) remains unknown. Although HMM showed advantages over the sliding window method in capturing the temporal fluctuations of DFC at a single time point in functional magnetic resonance imaging (fMRI) data, it is unclear whether HMM works well in revealing the time-varying functional connectivity of EEG data. In this study, the hidden Markov model (HMM) was introduced to DFC analysis of EEG data for the first time and was used to investigate the DFC features that can be used to assess 3D visual discomfort. The results indicated that state 2, with strong connections between electrodes, occurred more frequently in the early period, whereas state 4, with overall weak connections between electrodes, occurred more frequently in the late period for both visual comfort and discomfort stimuli. Moreover, the 3D visual discomfort stimuli caused subjects to stay in state 4 more frequently, especially in the later period, in contrast to the 3D visual comfort stimuli. The results suggest that the increasing occurrence of state 4 was possibly related to visual discomfort and that the occurrence frequency of state 4 may be used to assess visual discomfort.

## 1. Introduction

Stereoscopic displays have been widely applied to provide a vivid stereoscopic viewing experience to viewers. However, stereoscopic displays can induce visual discomfort due to the presence of many factors, such as excessive disparities, mismatch and crosstalk between left and right images, and accommodation–vergence conflict [1]. Therefore, the objective assessment of 3D visual discomfort is essential for evaluating stereoscopic display quality.

Among various objective assessment methods of 3D visual discomfort, the electroencephalogram (EEG) technique, which measures brain activities with high temporal resolution, has been used to reveal the neural features relevant to visual discomfort. Both the frequency features and the temporal features that were related to 3D visual discomfort were extracted from EEG signals. For the frequency domain analysis of EEG, the power of four wavebands, including alpha (α), beta (β), theta (θ), and delta (δ), changed with 3D visual discomfort [1,2,3,4,5,6]. For the temporal domain analysis of EEG, event-related potential (ERP) analysis found delayed P700 and P600 in 3D visual fatigue [2,7] and a significant correlation between 3D visual fatigue and P1 amplitude and N1, which originates from the posterior cingulate cortex [8]. Although these previous studies found important frequency and temporal features that can be used to assess 3D visual discomfort, the brain activity of each electrode was treated separately, and the functional connectivity (FC) between the electrodes was not considered.

Recently, it was demonstrated that FC between brain regions is not stationary and changes dynamically with time [9]. Dynamic functional connectivity studies have been widely performed using functional magnetic resonance imaging (fMRI) [10,11]. Among various DFC methods, the sliding window method is the simplest way to assess dynamic connectivity [11] and has been applied to EEG data [12,13,14]. However, the length of the sliding window can affect the performance of the sliding window method and reduce the temporal resolution. A useful alternative method to the sliding window method is the hidden Markov model (HMM), which has been demonstrated to be able to estimate DFC states at single time points without specifying window length from fMRI data [15,16,17]. Moreover, several studies applied HMM to EEG/MEG data to reveal DFC during the resting state [18] or the task state [19,20]. In contrast to the fMRI technique, which exhibits the sluggish nature of hemodynamic signals, EEG data capture the fast fluctuations of neural interactions and contain richer temporal dynamic information of neural activities than fMRI data. Therefore, HMM is potentially a powerful method to extract fast fluctuations of dynamic functional connectivity from EEG data.

This study aimed to introduce HMM to the DFC analysis of EEG data and investigated the temporal fluctuations of dynamic functional connectivity underlying 3D visual discomfort. Three-dimensional visual stimuli with different disparities were used to induce visual discomfort and visual comfort. The event-related potential (ERP) waves of visual discomfort and comfort were extracted from EEG data to remove simultaneously ongoing random brain processes. After the electrodes that showed significant differences in ERP components between visual discomfort and comfort conditions were selected, HMM was applied to the ERP waves of the selected electrodes to extract the dynamic brain states underlying the two conditions. It was demonstrated that dynamic FCs changed with development [21], aging [22], and training [23]. We hypothesized that a specific FC state relevant to visual discomfort existed and that the temporal property of DFC of the relevant state would change after 3D visual discomfort occurred and could be used to assess 3D visual discomfort.

## 2. Materials and Methods

The EEG data used in this study were collected from another study [24]. The experimental design of this study is briefly described.

### 2.1. Subjects

Forty-three participants, including 22 males and 21 females, aged 22 to 25 years, were recruited from Beijing Normal University. All participants had normal vision and were screened for stereo deficits using a stereo test. The experiment was approved by the ethics committee of Beijing Normal University. All subjects provided written informed consent prior to the experiment.

### 2.2. Visual Stimuli

A 3D LCD with LED Backlight (LG D2343p, 1920 × 1080 pixels) was used to display the 3D visual stimuli. The stereoscopic display was 1 m away from subjects. The disparity levels of the 3D visual stimuli ranged from ±0.1° to ±1°. The 3D visual stimuli were divided into two sets: comfort and discomfort stimuli based on the subjective assessment in the behavior experiment prior to the EEG experiment. The comfortable 3D stimuli had binocular disparities equal to +0.2°, +0.4°, −0.3°, −0.5°, and −0.7°. The discomfort 3D stimuli had binocular disparities equal to +0.6°, +0.8°, +1.0°, −0.9°, and −1.0°. The 2D visual shape sample and background that were used to generate the 3D visual stimuli with different disparity levels are presented in Figure S1 of the Supplementary Materials.

### 2.3. EEG Experiment

The EEG experiment was performed in a dedicated and quiet room with a constant room temperature. All participants were seated in a comfortable chair during the experiment. The EEG calibration procedure was performed by applying a known rectangular wave to all channels of the EEG instrument to correct the accuracy of the signal amplitude. The EEG signals were recorded at a sampling rate of 512 Hz from 128 scalp electrodes following 10–20 conventions with an EGI system. The resistance of each electrode was guaranteed to range from 0 to 50 KΩ. The schematic setup of the EEG experiment is presented in Figure S2 of the Supplementary Materials. For each trial, a cross was presented for 2 s. After the cross stimulus disappeared, 3D visual stimuli were presented for 3 s, and a blank screen was presented for 3 s following the 3D stimuli. The subjects were required to judge whether the 3D stimulus was in front of or behind the screen when the 3D stimulus was presented. If the subjects judged the stimulus being in front of the screen, they were required to press the left arrow key. Otherwise, they were required to press the right arrow key.

### 2.4. EEG Data Preprocessing

EEGlab (http://sccn.ucsd.edu/eeglab (accessed on 1 September 2019)) in MATLAB R2017b was used to preprocess the EEG data. The EEG data were rereferenced to the average of all the electrodes and filtered by bandpass filtering (0.1–32 Hz). After filtering, the EEG data were segmented in epochs of 3.2 s, starting from 200 ms before stimulus onset to 3 s after stimulus onset. All epochs were concatenated and baseline corrected. Bad channels were detected and interpolated, and large artifact time points were rejected. Independent component analysis (ICA) was performed for artifact removal. Among the 43 subjects, 6 subjects were removed, because they had numerous bad channels and contained large noises throughout the entire experiment. Therefore, a total of 37 subjects were used in the following analysis.

### 2.5. Electrode Selection

For each electrode of each subject, the comfort/discomfort ERP waves were obtained by averaging EEG epochs across trials related to the comfort/discomfort conditions. In addition, the ERP mean amplitudes in 4 time windows (i.e., 90–130, 100–140, 180–220, and 280–320 ms) that corresponded to the 4 ERP components (i.e., P1, early N1, late N1, and P3) were computed for each condition and electrode. Paired *t*-tests were performed to detect the electrodes that showed significant differences between the visual comfort and discomfort conditions (*p* < 0.05, uncorrected).

### 2.6. Hidden Markov Modeling

The HMM assumes that the discrete-time stochastic process that produces the observed time series Y depends on a Markov process [25]. The Markov process has a finite number of states. For the time series with T time points, let the time series Y = {Y_1_, Y_2_, …, Y_T_} and the hidden state sequence S = {S_1_, S_2_, …, S_T_}. The vector Y_i_ represents ERP signals of M electrodes at the ith time point. It is assumed that each Y_i_ satisfies a multivariate Gaussian distribution P(Y_i_|S_i_ = k) ~ N(μ_k_, Ø_k_). Here, P(Y_i_|S_i_ = k) represents the probability that Y_i_ can be observed under state k at time point i. The parameter μ_k_ is the mean vector with the size M × 1, and Ø_k_ is the covariance matrix with the size M × M. M is the number of electrodes. The covariance matrix Ø_k_ represents the functional connectivity pattern between the M electrodes for state k. The Viterbi path was computed to determine the hidden state to which Yi belonged using the Viterbi algorithm.

### 2.7. Temporal Property Analysis of DFC

HMM was applied to the ERP signals of all the selected electrodes of all subjects for the visual comfort and visual discomfort conditions, separately. The HMM-MAR toolbox (https://github.com/OHBA-analysis/HMM-MAR (accessed on 1 May 2020)) was used to perform HMM. The number of states was set to four. For the visual comfort/discomfort condition, the ERP signals of all selected electrodes were concatenated across all 37 subjects. HMM was applied to the concatenated ERP signals of visual comfort/discomfort to estimate the FC patterns and state time series of the FC states. The number of the brain states were set to 4. We ran the HMM 20 times and chose the result with minimum free energy. The mean dwell time and fraction of time were calculated for each state of each subject. The mean dwell time of a DFC state was calculated as the average number of consecutive time points corresponding to the state. The fraction of time of a state was calculated as the proportion of time points corresponding to the state. For the mean dwell time/fraction of time of each state, paired *t*-tests were applied to all subjects to test the differences between the visual comfort and discomfort conditions.

## 3. Results

### 3.1. Electrode Selection

**Figure 1:**
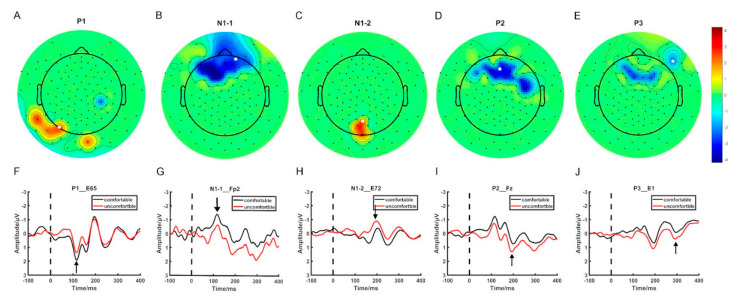
The spatial distribution of the selected electrodes. (**A**–**E**) The topographic map of the ERP components showing significant differences between visual comfort and discomfort. (**F**–**J**) The EPR waveforms of the selected electrodes.

Figure 1 displays the electrodes that showed significant differences in ERP mean amplitude between the visual comfort and discomfort conditions for all four time windows. In Figure 1, the scalp was divided into four regions: the frontal region (F), the temporal region (T), the parietal region (P), and the occipital region (O). Among all 42 selected electrodes, 30 electrodes were located in the F region, 5 electrodes in the P region, 4 electrodes in the T region, and 3 electrodes in the O region. Most of the selected electrodes were located in the frontal region.

### 3.2. Temporal Property Analysis of DFC

**Figure 2:**
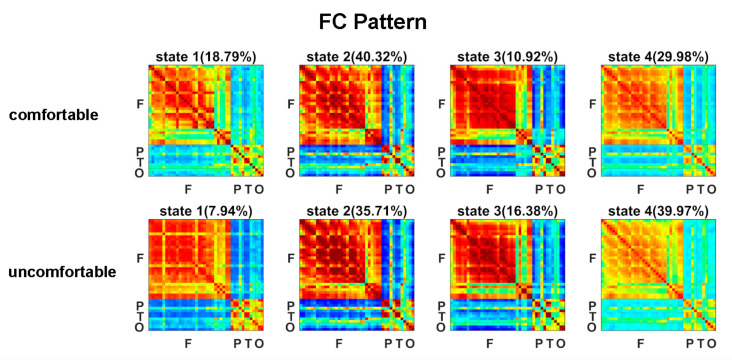
FC patterns of the four brain states for the visual comfort and discomfort conditions.

**Figure 3:**
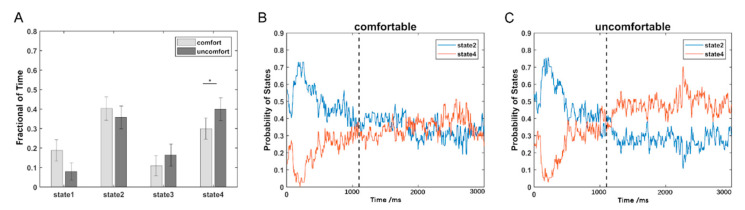
The temporal property comparisons of DFC: (**A**) comparison of the fraction of time between the visual comfort and discomfort conditions; (**B**) the probability variations of state 2 and state 4 with the time points for the visual comfort condition; (**C**) the probability variations of state 2 and state 4 with the time points for the visual discomfort condition. The star * represents *p* < 0.05.

The estimated FC patterns of the four states for visual comfort and discomfort are presented in Figure 2. The four FC patterns of the visual comfort condition were similar to those of the visual discomfort condition. The mean fractions of time of the four states are presented in Figure 3A. Among the four states, states 2 and 4 had the highest fraction of time for both the visual comfort and discomfort conditions. For state 4, visual comfort showed a significantly higher fraction of time than visual discomfort (T(1,36) = −3.1764, *p* = 0.0031 < 0.001). The mean dwell time of the four states did not significantly differ between visual comfort and visual discomfort.

Because states 2 and 4 were the two dominant states, the probabilities of the two states at each time point are shown in Figure 3B for visual comfort and Figure 3C for visual discomfort. In contrast to state 4, state 2 showed a higher probability in the early period (1100 ms) for both the visual comfort and discomfort conditions and a lower probability in the later period (>1100 ms) for visual discomfort. Moreover, states 2 and 4 showed similar probabilities at the later period (>1100 ms) for visual comfort.

**Figure 4:**
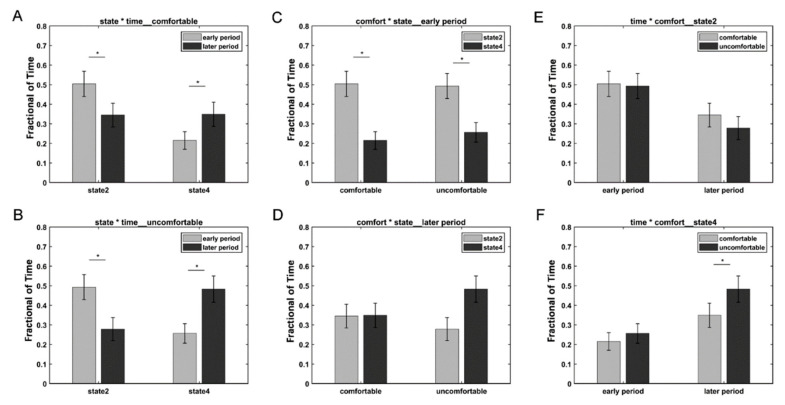
Results of the simple effects of the period (**A**,**B**), state (**C**,**D**), and condition (**E**,**F**). The star * represents *p* < 0.05.

We further divided the whole time points into two periods: the early period (1100 ms) and the later period (>1100 ms) based on the different variation trends of states 2 and 4. A three-way repeated-measures ANOVA test using the condition (i.e., comfort and discomfort), state (i.e., states 2 and 4), and period (i.e., early and later) as the three within-subject main factors was performed on the fraction of time of 34 subjects in SPSS Statistics 20.0. Three subjects were excluded from the three-way repeated-measures ANOVA test, because they did not exhibit state 2 or state 4. A significant three-way interaction was observed among the condition, state, and period (F(1,33) = 4.748, *p* = 0.037 < 0.05). Simple tests were performed to compare the effect of different levels of a factor at a particular level of a second factor within levels of a third factor. Figure 4 shows the comparison of different simple effects. For both the comfort and discomfort conditions, the simple effects of period revealed that the fraction of time of the early period was significantly greater in state 2 (comfort: F(1,33) = 28.3, *p* < 0.001; discomfort: F(1,33) = 31.42, *p* < 0.001) and significantly lower in state 4 (comfort: F(1,33) = 22.08, *p* < 0.001; discomfort: F(1,33) = 37.89, *p* < 0.001) compared with the later period. For the early period, the simple effects of the state revealed that the fraction of time of state 2 was significantly greater than that of state 4 in both the comfort (F(1,33) = 11.14, *p* = 0.002 < 0.01) and discomfort conditions (F(1,33) = 6.81, *p* = 0.014 < 0.05). For state 4, the simple effects of the condition revealed that the visual discomfort condition produced a significantly higher fraction of time than the visual comfort condition (F(1,33) = 14.77, *p* = 0.001).

## 4. Discussion

This study investigated the temporal fluctuations of DFC relevant to 3D visual discomfort by introducing the HMM to EEG data. We found that the subjects tended to more frequently remain in state 4, with overall weak connections, when they viewed the 3D discomfort stimuli compared to 3D comfort stimuli. State 2, with overall strong connections, occurred more frequently in the early period, whereas state 4 occurred more frequently in the late period for both the visual comfort and discomfort stimuli. In contrast to the visual comfort stimuli, the visual discomfort stimuli induced a greater occurrence of state 4 in the later period. These results may suggest that state 4 is possibly relevant to 3D visual discomfort and that the increasing frequency of the occurrence of state 4 may imply visual discomfort when subjects view 3D images.

Previous fMRI studies reported that 3D visual discomfort could change activation in the frontal, parietal, temporal, and occipital regions [26]. In this study, the electrodes that showed significant differences in ERP components between the visual comfort and discomfort stimuli were also located in the frontal, parietal temporal, and occipital regions, which was consistent with the findings of the previous study. Moreover, the frontal region contained the most selected electrodes, which may indicate that the frontal regions play an important role in processing 3D visual discomfort stimuli.

Among the four states, the fraction of time of state 2 and state 4 were the highest (see Figure 3A), potentially indicating that these two states are dominant in viewing 3D images. In contrast to state 2, state 4 showed weaker positive connectivity between electrodes within the frontal, parietal, temporal, and occipital regions (see Figure 2). The weaker interactions in state 4 and stronger connections in state 2 may suggest that the subjects could process the 3D visual stimuli more actively in state 2 compared with state 4.

In contrast to visual comfort stimuli, 3D visual discomfort stimuli induced a greater occurrence of state 4. Among the four states, state 4 had the weakest connections between electrodes. The overall weak connections in state 4 could indicate that the brain regions did not transfer information to each other actively and efficiently in this state. Moreover, the dynamic state with weaker connectivity between and within networks was more frequently observed during rest than during tasks [27]. Therefore, it could be inferred that state 4 was possibly related to relaxation [21]. When the subjects viewed 3D visual discomfort stimuli, the feeling of discomfort tended to induce eye fatigue, and the subjects were more willing to stay in state 4 to relax than in state 2 to actively process 3D visual stimuli. Thus, more occurrences of state 4 were observed when viewing the 3D visual discomfort stimuli versus comfort stimuli.

Moreover, the occurrences of states 2 and 4 showed different dynamic variations with time (see Figure 4). For both the 3D visual comfort and discomfort stimuli, state 2 occurred more frequently in the early period compared with the later period, whereas state 4 occurred more frequently in the later period than in the early period. In the early period, subjects tended to stay more frequently in state 2 compared with state 4 for both visual comfort and discomfort stimuli. The results suggested that neither visual comfort nor discomfort stimuli induced obvious visual discomfort and that subjects could actively process 3D visual stimuli in the early period. In the later period, the occurrence of state 4 increased, whereas the occurrence of state 2 decreased for the two conditions. This finding may indicate that longer viewing comfort and discomfort 3D stimuli could cause subjects to be more likely to stay in state 4 to relax their eyes. Moreover, the visual discomfort stimuli caused subjects to spend significantly more time in state 4 than state 2, whereas the visual comfort stimuli did not induce significant differences between states 2 and 4 in the later period. In contrast to the 3D visual comfort stimuli, the 3D visual discomfort stimuli could induce stronger visual fatigue and discomfort and cause the subjects to be more likely to relax in the later period. Therefore, state 4 was more frequently observed in the later period when the subjects viewed the 3D visual discomfort stimuli compared to the comfort stimuli. Based on the above results, it can be inferred that the occurrence of state 4 was possibly relevant to visual discomfort, and the increase in state 4 might suggest the emergence of visual discomfort. As a result, the fraction of time spent in state 4 could be used to assess 3D visual discomfort when subjects viewed 3D visual stimuli. Moreover, the electrodes showing significant ERP differences between conditions were selected by the ERP components that occurred at the early stage, while the significant differences in brain states between the two conditions occurred at the later stage. The ERP components reflected the energy variation of each electrode with time. In contrast, the brain state revealed by HMM reflected the interactions between different electrodes. Thus, the ERP analysis and DFC analysis revealed different information on the EEG data from different aspects. The results may suggest that the energy variations of individual electrodes appeared much earlier than the variations in DFC between different electrodes.

Moreover, there were some limitations in this study. Firstly, it should be noted that the physiological processes that captured the visual discomfort were not recorded in the EEG experiment. It is necessary to simultaneously record the physiological processes and neural activities in the EEG experiment so that the variations in the pattern of neural activity can be compared with that of the physiological process to exclude the variation induced by noises in future studies. Secondly, the HMM with Gaussian observation model may not be optimal for EEG raw data. The covariance matrix of the Gaussian observation model reflected a zero-lagged relationship between the electrodes, which may be affected by spurious connectivity related to the volume conductor problem.

## 5. Conclusions

In this study, the HMM was applied to EEG data to investigate the temporal changes in DFC induced by 3D visual discomfort. The results indicated that the subjects were possibly more willing to remain in state 2, with strong connections, to process the 3D visual stimuli actively in the early period. When the subjects viewed 3D visual stimuli longer, the occurrence of state 4, with weak connections, increased and that of state 2 decreased in the later period. More importantly, the 3D visual discomfort stimuli induced a significantly greater occurrence of state 4 in the later period in contrast to the 3D visual comfort stimuli. Our results may suggest that state 4 might be relevant to visual discomfort and that the fraction of time spent in state 4 could be used to assess 3D visual discomfort.

## Data Availability

The original data are available upon request from the author.

## Supplementary Materials

The following are available online at https://www.mdpi.com/article/10.3390/brainsci12070937/s1, Figure S1: One 2D shape sample (a) and the background (b) that were used to generate 3D visual stimuli; Figure S2: The schematic setup of the EEG experiment: (a) the experiment process, (b) the experiment paradigm.
