# Supplementary material for: Assessment of 3D Visual Discomfort Based on Dynamic Functional Connectivity Analysis with HMM in EEG
Source: Brain Sci. 2022 Jul 18;12(7):937. doi: 10.3390/brainsci12070937 (PMC9313185; doi:10.3390/brainsci12070937)
Supplement: Supplementary file 1 [file brainsci-12-00937-s001.zip › brainsci-1687634-Supplementary Materials.pdf]

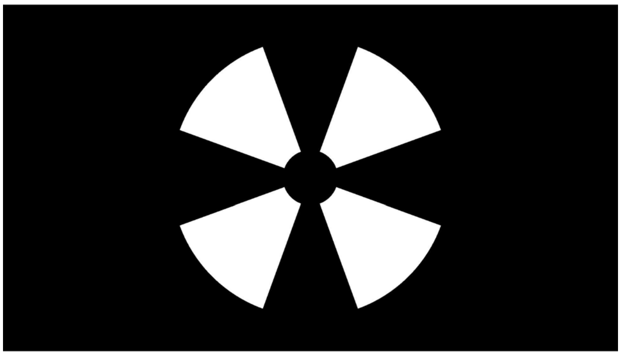

(a)

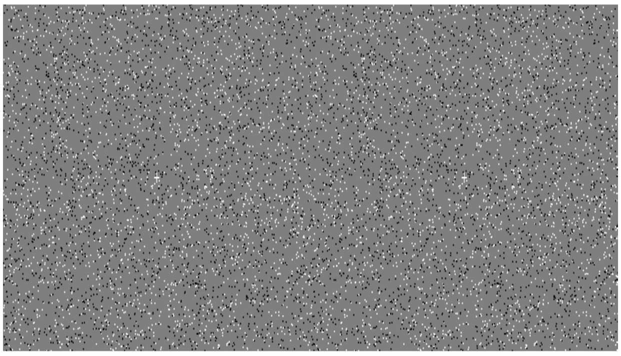

(b)

Figure S1. One 2D shape sample (a) and the background (b) that were used to generate 3D visual stimuli.

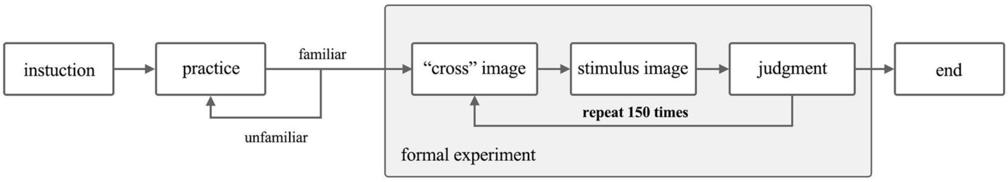

(a)

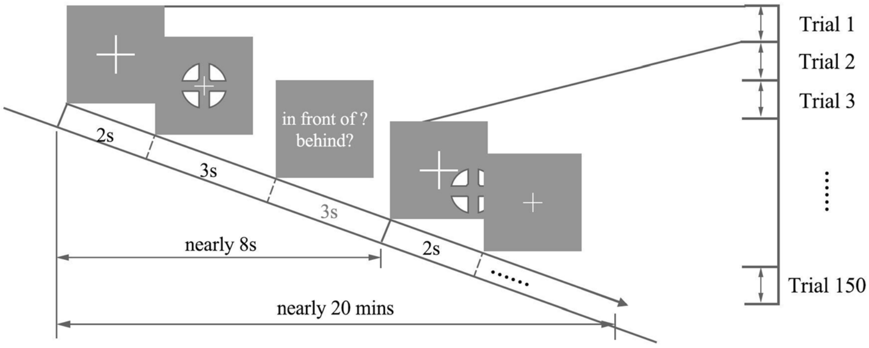

(b)

Figure S2. The schematic setup of the EEG experiment: (a) the experiment process, (b) the experiment paradigm.
